# Association Between Early Joint Awareness and Falls After Total Hip Arthroplasty

**DOI:** 10.1016/j.artd.2026.102151

**Published:** 2026-11-04

**Authors:** Osamu Wada, Koutatsu Nagai, Masataka Igeta, Akiko Mori, Kiyonori Mizuno

**Affiliations:** a Graduate School of Rehabilitation Science, Hyogo Medical University, Kobe, Hyogo, Japan; b Department of Rehabilitation, Anshin Hospital, Kobe, Hyogo, Japan; c Department of Physical Therapy, School of Rehabilitation, Hyogo Medical University, Kobe, Hyogo, Japan; d Department of Biostatistics, Hyogo Medical University, Nishinomiya, Hyogo, Japan; e Department of Orthopaedics, Anshin Hospital, Kobe, Hyogo, Japan

**Keywords:** Fall, Forgotten joint score, Patient-reported outcome measure, Rehabilitation, Total hip arthroplasty

## Abstract

**Background:**

The Forgotten Joint Score-12 (FJS-12) is a patient-reported outcome measure of joint awareness after total hip arthroplasty (THA). Falls after THA remain clinically important. However, longitudinal evidence regarding the associations among early FJS-12, subsequent falls, and later recovery of joint unawareness remains limited.

**Methods:**

This retrospective cohort study used a prospectively maintained institutional database from a single Japanese institution. Consecutive patients who underwent primary THA between January 2021 and December 2023 were included. FJS-12 was assessed at 3 and 12 months postoperatively, and falls occurring between 3 and 12 months postoperatively were identified at the 12-month visit based on patients’ self-report over this 9-month interval. Modified Poisson regression was used to examine the association between FJS at 3 months and subsequent falls. Multiple linear regression was used to examine the association between fall occurrence and FJS at 12 months, adjusting for FJS at 3 months.

**Results:**

Overall, 623 patients were analyzed (mean age 70.8 years; 85.2% female). Ninety-nine patients (15.9%) experienced at least 1 fall between 3 and 12 months postoperatively. Lower FJS at 3 months was associated with a higher risk of subsequent falls. Fall occurrence was associated with lower FJS at 12 months, independent of FJS at 3 months and other covariates.

**Conclusions:**

Early postoperative joint awareness was associated with subsequent falls after THA, and falls were associated with lower FJS at 12 months. The FJS-12 may provide additional patient-reported information when assessing postoperative fall risk.

## Introduction

Total hip arthroplasty (THA) is a widely performed procedure that provides substantial improvement in pain relief, mobility, and quality of life in patients with advanced hip pathologies, particularly osteoarthritis [1]. With an aging population, the global demand for THA continues to increase [2]. Accordingly, interest has expanded beyond traditional clinical outcomes to include patient-reported outcomes that capture functional recovery and joint awareness. In this context, the Forgotten Joint Score (FJS) has emerged as a validated instrument measuring the extent to which patients can "forget" their artificial joint during daily activities [3,4], thereby reflecting greater functional recovery and higher patient satisfaction [5].

In addition to joint awareness as a qualitative aspect of recovery, postoperative falls remain a major concern. Approximately 25–36% of patients experience at least 1 fall within the first postoperative year [6], with a proportion of these falls resulting in fractures or other serious injuries [7]. Falls may lead to fear of falling and reduced confidence, potentially limiting activity and compromising functional recovery [[8], [9], [10]].

A lower FJS-12, indicating greater awareness of the operated hip during daily activities, may be associated with fall risk after THA, as it may reflect persistent discomfort and reduced functional confidence, potentially leading to a cautious gait and greater attentional demands during walking [11]. Related to this concept, recent evidence suggests that motor-cognitive deficits may present after THA and may be relevant to postoperative gait and balance [12]. From a THA-specific perspective, THA surgery involves surgical intervention around periarticular structures that may contain mechanoreceptors relevant to hip proprioception [13]. In addition, impairments in balance and postural control may persist after THA [14]. These factors may help explain why greater awareness of the operated hip during daily activities, as reflected by lower FJS-12, could be related to a subsequent increase in fall occurrence. However, longitudinal evidence linking early postoperative FJS-12 to subsequent falls remains limited. In addition, whether postoperative falls are associated with later joint awareness during recovery has not been well described.

Therefore, the primary aim was to investigate whether lower FJS in the early phase was associated with a higher incidence of falls during the subsequent postoperative period after THA. We hypothesized that lower FJS in the early phase would be associated with a higher risk of subsequent falls. The secondary aim was to examine whether postoperative falls were associated with FJS at 12 months. We hypothesized that patients who experienced falls during this period would have lower FJS at 12 months.

## Material and methods

### Design, setting, and participants

This study was a retrospective analysis of a prospectively maintained institutional database at a single institution in Japan. We included consecutive patients who underwent primary THA performed by 1 of 5 arthroplasty surgeons between January 1, 2021, and December 31, 2023. Surgical approaches were either the direct anterior approach or the anterolateral supine approach. Patients were excluded if they had undergone hip or knee arthroplasty within the past year, had cognitive impairment, intraoperative complications, neurological disorders affecting gait and balance control (eg, cerebrovascular disease), or nonelective indications (eg, trauma or infection).

Clinical and functional outcomes were collected prospectively at scheduled follow-up visits, whereas fall incidents between 3 and 12 months postoperatively were retrospectively ascertained through self-reports at the 12-month evaluation. No a priori power calculation was performed because this study was a retrospective analysis of a prospectively maintained institutional database. The study size was determined by including all eligible patients who underwent primary THA during the study period and had complete data for the variables included in the analyses.

This longitudinal study was approved by the institutional review board of our institution (approval no. 5292). The current analysis was conducted retrospectively using these data and involved no additional procedures or burden to participants.

### FJS-12 questionnaire

Joint awareness was evaluated using the FJS-12, a validated joint-specific patient-reported outcome measure. The FJS-12 consists of 12 items rated on a 5-point Likert scale, converted to a total score ranging from 0 to 100, with higher scores indicating less awareness of the artificial joint and better functional outcomes. In this study, the FJS-12 was administered at 3 and 12 months postoperatively to capture changes in joint awareness over time. The 3-month postoperative period represents a clinically meaningful phase when most patients regain community ambulation and can evaluate joint awareness during daily activities [15]. The validated Japanese version of the FJS-12 was used [16].

### Postoperative fall assessment

A fall was defined as "an unexpected event in which the participant comes to rest on the ground, floor, or a lower level, not as a result of a major intrinsic event (eg, fainting, seizure, or stroke) or an overwhelming external hazard" [17]. At the 12-month postoperative visit, patients were asked whether they had experienced any falls between 3 and 12 months after surgery, and the number of falls during this period was recorded. Falls occurring before 3 months postoperatively were not included in the primary analysis because the primary exposure was the FJS-12 assessed at 3 months. Including falls that occurred before the exposure assessment would have disrupted the temporal sequence between early postoperative FJS-12 and subsequent fall occurrence.

### Confounding variables assessment

Potential confounders included age [18], sex [18], body mass index [19], history of depression [20], contralateral hip condition [21], pain intensity [22], quadriceps muscle strength [23], and gait abnormality [24]. Postoperative variables were assessed at 3 months postoperatively.

A history of depression was confirmed from medical records at the time of surgery. The condition of the contralateral hip was assessed using standardized anteroposterior pelvic radiographs. A trained observer evaluated osteoarthritis according to the Kellgren–Lawrence grading system [25] and confirmed any history of contralateral THA. Patients with grade III or IV osteoarthritis were classified as having advanced disease. Intrarater reliability, which was evaluated by re-examining a random subset of 100 radiographs after 2 weeks, was excellent (κ = 0.86). Postoperative hip pain intensity was measured using a numeric rating scale (0–10), where 0 indicates no pain, and 10 indicates the worst imaginable pain [26]. Quadriceps muscle strength on the operated side was assessed using a handheld dynamometer with a stabilization belt, measuring peak isometric knee extension torque at 90° hip and knee flexion. The highest value from two maximal trials was normalized to body weight (Nm/kg) [27]. Gait abnormalities were evaluated at 3 months postoperatively while patients walked at a comfortable speed following established procedures [24]. Well-trained physical therapists used an observational scale, and patients were classified as having a gait abnormality if moderate or greater deviations (eg, pronounced pelvic motion or lateral trunk sway) were observed. This method has demonstrated excellent intrarater and inter-rater reliability [28].

### Data analyses

All participants included in the analytical cohort had complete data for the variables included in the analyses. Baseline characteristics are presented as the mean ± standard deviation for continuous variables and as number (percentage) for categorical variables.

Two complementary analyses were conducted. In the primary analysis, the association between the FJS-12 at 3 months postoperatively and fall occurrence between 3 and 12 months after THA was examined using modified Poisson regression to estimate the relative risks with Huber–White sandwich robust standard errors. To provide an absolute measure of association, adjusted predicted risks were estimated from the modified Poisson regression model. For this calculation, the 3-month FJS-12 was set to the cohort mean value and to a value 10 points below the cohort mean, while all other covariates were kept at their observed values for each patient. The predicted risks were then averaged across the study cohort, and the adjusted absolute risk difference was calculated as the difference between these averaged predicted risks. As a sensitivity analysis, the same model was applied using the number of falls during this period as the outcome.

In the secondary analysis, multiple linear regression was used to examine whether fall occurrence was associated with FJS at 12 months after adjustment for FJS at 3 months. The 12-month FJS served as the dependent variable, fall occurrence between 3 and 12 months as the independent variable, and the 3-month FJS was included as a covariate. The Huber–White sandwich robust standard error estimator was used. All models adjusted for the potential confounders described above.

All statistical analyses were performed using Stata/MP version 19 (StataCorp, College Station, TX, USA). A two-sided *P* < .05 was considered statistically significant.

## Results

### Baseline characteristics

Figure 1 presents a flowchart of the participant selection process. Overall, 858 patients who underwent unilateral THA and completed the 3-month postoperative evaluation were initially considered. After applying the exclusion criteria, 808 patients remained eligible. Of these, 185 patients were excluded because of contralateral hip surgery, worsening symptoms in joints other than the contralateral hip, relocation, or loss to follow-up. Finally, 623 patients were included in the final analysis. Supplementary Table 1 compares the characteristics of the included patients, patients lost to follow-up, and patients excluded for other reasons. Some between-group differences were observed, particularly in age and contralateral hip condition. Table 1 summarizes the characteristics of the final study cohort. The mean patient age was 70.8 years (range, 42–95 years), and 85.2% were female. In total, 99 patients experienced at least 1 fall during the study period, representing 15.9% of the total participants. No fractures or other serious fall-related injuries were documented among patients who experienced falls. The baseline characteristics stratified by the number of falls among patients who experienced at least 1 fall are presented in Supplementary Table 2. Of the total participants, 86 (13.8%) experienced 1 fall, 11 (1.8%) experienced 2 falls, and 2 (0.3%) experienced 3 falls.

**Figure 1:**
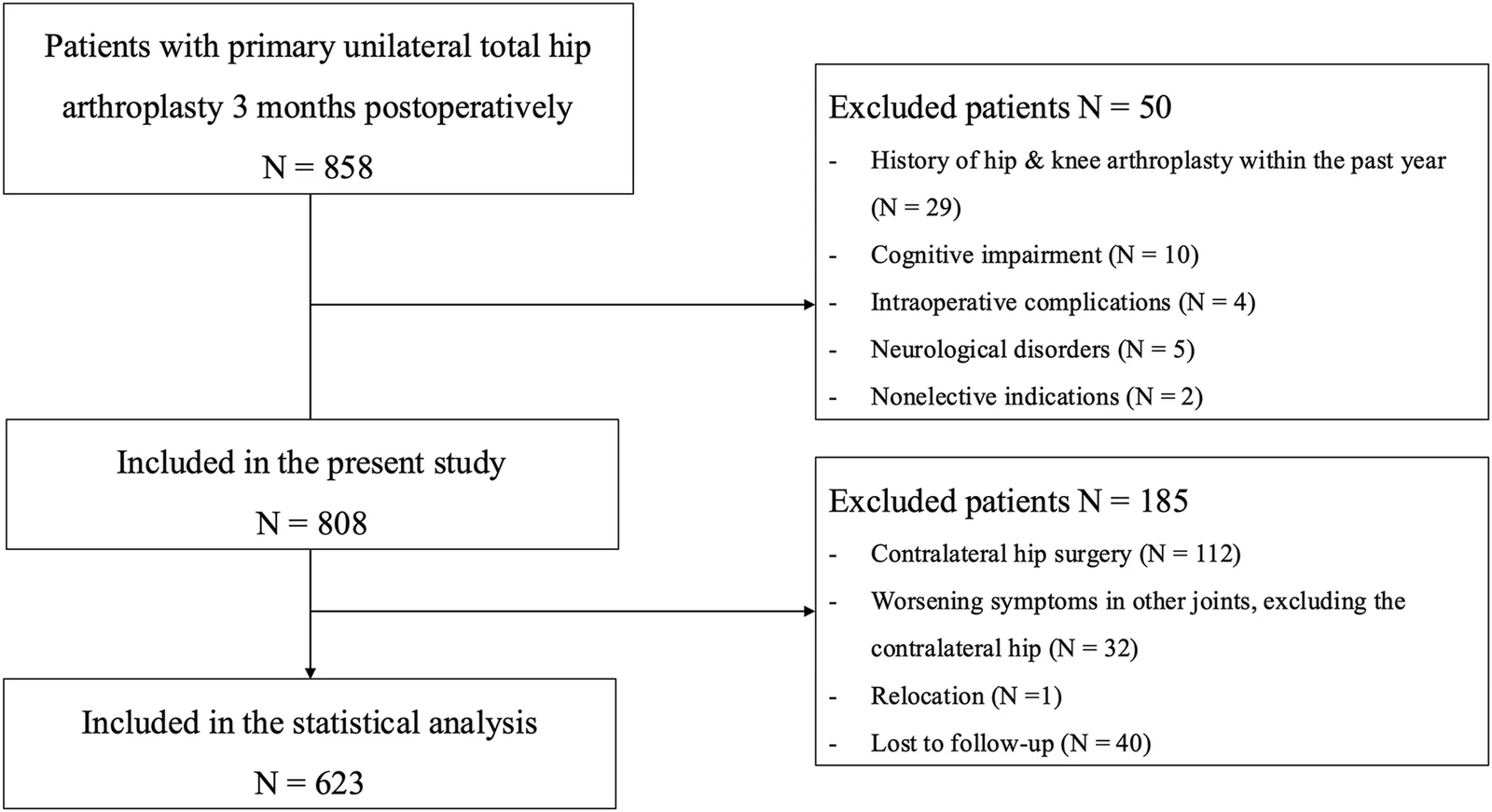
Patient flow.

**Table 1:** Characteristics of patients with and without falls.

Characteristics	All participants		No-fall group		Fall group		SMD
	N = 623		N = 524		N = 99		
	Mean (SD)	Median [IQR]	Mean (SD)	Median [IQR]	Mean (SD)	Median [IQR]	
Age, year	70.8 (9.4)	71.0 [65.0–77.0]	70.6 (9.3)	71.0 [65.0–77.0]	72.2 (9.9)	74.0 [65.0–80.0]	0.17
Sex							0.13
Men	92 (14.8)		81 (15.5)		11 (11.1)		
Women	531 (85.2)		443 (84.5)		88 (88.9)		
BMI	23.9 (3.9)	23.5 [21.1–26.1]	23.8 (3.9)	23.4 [21.0–26.1]	24.4 (4.0)	23.7 [21.9–26.4]	0.16
Hip condition in the contralateral side							
Total hip arthroplasty	59 (9.4)		50 (9.5)		9 (9.1)		0.02
Osteoarthritis, no	492 (79.0)		414 (79.0)		78 (78.8)		0.01
Osteoarthritis, yes	72 (11.6)		60 (11.5)		12 (12.1)		0.02
Knee arthroplasty, yes	28 (4.5)		25 (4.8)		3 (3.0)		0.09
Depression, yes	9 (1.4)		7 (1.3)		2 (2.0)		0.05
Pain, point	1.3 (1.8)	0.0 [0.0–2.0]	1.3 (1.8)	0.0 [0.0–2.0]	1.6 (1.8)	1.0 [0.0–3.0]	0.14
Quadriceps muscle strength, Nm/kg	1.1 (0.4)	1.0 [0.8–1.3]	1.1 (0.3)	1.0 [0.9–1.3]	1.0 (0.4)	1.0 [0.7–1.2]	0.39
Gait abnormality, yes	44 (7.1)		34 (6.5)		10 (10.1)		0.13
FJS-12, point							
3 months	60.8 (23.4)	62.5 [45.8–77.3]	62.2 (23.1)	63.9 [50.0–79.2]	53.5 (24.2)	54.5 [36.4–72.5]	0.37
12 months	72.1 (23.3)	75.0 [58.3–91.7]	75.3 (21.7)	79.2 [63.6–93.8]	55.3 (24.0)	54.2 [37.5–70.8]	0.87

Values are presented as mean (SD), median [IQR], or n (%). SMDs were calculated as the difference between the falls and no-falls groups. An absolute SMD ≥0.10 was used as a descriptive reference for between-group differences.

The FJS-12 ranged from 0 to 100, with higher scores indicating lower awareness of the joint during daily activities.

IQR, interquartile range; SD, standard deviation; SMD, standardized mean difference.

### Association between the FJS at 3 months and subsequent falls (primary analysis)

A modified Poisson regression model showed that lower FJS at 3 months postoperatively was significantly associated with a higher incidence of subsequent falls (relative risk per 10-point decrease, 1.12; 95% confidence interval [CI], 1.02–1.21; *P* = .013) (Table 2). This association remained significant in the sensitivity analysis in which the number of falls was used as the outcome variable (incidence rate ratio per 10-point decrease, 1.16; 95% CI, 1.06–1.26; *P* < .001) (Supplementary Table 3). To provide clinical context, the adjusted predicted risk of subsequent falls was 15.2% at the mean FJS-12 score at 3 months of 60.8 points and 17.0% at an FJS-12 score 10 points lower, corresponding to an adjusted absolute risk difference of 1.8 percentage points (95% CI, 0.4–3.2; *P* = .012).

**Table 2:** Association between fall occurrence between 3 months and 12 months postoperatively and the FJS-12 at 3 months postoperatively.

Variables	RR	95% confidence interval		*P* value
		Lower	Upper	
FJS-12 at 3 months postoperatively, per 10-point decrease	1.12	1.02	1.21	.013
Age, year	1.01	0.99	1.03	.240
Sex, men	0.87	0.49	1.57	.652
BMI	1.01	0.97	1.06	.517
Depression, yes	1.76	0.58	5.39	.322
THA in the contralateral side, yes	0.89	0.47	1.68	.717
OA in the contralateral side, yes	0.96	0.55	1.66	.879
Pain at 3 months postoperatively	0.97	0.87	1.08	.603
Quadriceps muscle strength at 3 months postoperatively	0.50	0.25	1.01	.055
Gait abnormality, yes	1.18	0.65	2.17	.584

BMI, body mass index; RR, relative risk; OA, osteoarthritis.

Covariates were included in the model for adjustment purposes and were not the primary variables of interest.

### Association between fall occurrence and the FJS at 12 months (secondary analysis)

Multiple linear regression analysis showed that fall occurrence between 3 and 12 months was significantly associated with lower FJS at 12 months (β = −14.07 points; 95% CI, −18.67–−9.48; *P* < .001), even after adjustment for FJS at 3 months and other potential confounders. Among the covariates, older age (β = −0.18; *P* = .035), history of depression (β = −12.57; *P* = .032), and quadriceps muscle strength at 3 months (β = 7.00; *P* = .003) were significantly associated with FJS at 12 months. FJS at 3 months remained a strong predictor of FJS at 12 months (β = 0.54; 95% CI, 0.47–0.61; *P* < .001) (Table 3).

**Table 3:** Association between postoperative fall occurrence (3–12 months) and the FJS-12 at 12 months after THA.

Variables	B (coefficient)	95% confidence interval		*P* value
		Lower	Upper	
Fall occurrence	−14.07	−18.67	−9.48	<.001
FJS-12 at 3 months postoperatively, point	0.54	0.47	0.61	<.001
Age, year	−0.18	−0.35	−0.01	.035
Sex, men	−1.40	−5.75	2.94	.526
BMI	−0.02	−0.41	0.36	.907
Depression, yes	−12.57	−24.10	−1.05	.032
THA in the contralateral side, yes	0.51	−4.33	5.36	.835
OA in the contralateral side, yes	0.04	−4.33	4.41	.987
Pain at 3 months postoperatively	0.01	−0.96	0.97	.986
Quadriceps muscle strength at 3 months postoperatively	7.00	2.33	11.67	.003
Gait abnormality, yes	1.13	−5.42	7.68	.735

BMI, body mass index; OA, osteoarthritis.

Adjusted R^2^ = 0.42.

Covariates other than FJS-12 at 3 months were included in the model for adjustment purposes and were not the primary variables of interest.

## Discussion

In this study, we examined the longitudinal association between joint awareness and falls after THA. Our findings supported the hypothesis that lower FJS at 3 months postoperatively was associated with a higher risk of subsequent falls between 3 and 12 months. Although the relative association between lower FJS at 3 months and subsequent falls was statistically significant, the corresponding absolute risk difference was modest. These findings suggested that FJS should be interpreted as a patient-reported measure that may provide additional information when considered alongside established fall risk factors. Additionally, we found that falls during this period were associated with lower FJS at 12 months, even after adjustment for FJS at 3 months. Taken together, these results suggest a longitudinal association between joint awareness and falls during the postoperative recovery period. Although causality cannot be established, these findings suggest that early postoperative FJS may provide clinically relevant information about subsequent fall risk, and that falls may identify a subgroup of patients with lower FJS at 12 months after THA.

The FJS values observed in this study (60.8 points at 3 months and 72.1 points at 12 months postoperatively) were generally consistent with those reported in previous THA studies [[29], [30], [31], [32]], suggesting that the level of joint awareness in this cohort was within the expected range. In contrast, the incidence of falls in this study (15.9%) was lower than that reported in previous studies (approximately 25–36%) [6]. This difference may be partly explained by the exclusion of falls occurring within the first 3 months postoperatively, a period during which fall risk may be higher.

Several mechanisms may explain the association between lower FJS and subsequent falls. Lower FJS reflects greater awareness of the operated hip during daily activities and may indicate incomplete functional recovery [33]. Under these conditions, patients may allocate increased attention to the operated hip during walking and other functional tasks, reducing gait automaticity and increasing cognitive demands. Impaired motor automaticity and greater reliance on attentional resources during gait have been reported in individuals with musculoskeletal impairments and may contribute to postural instability during everyday activities [34]. In addition, altered proprioceptive input may also be relevant after THA. THA surgery may involve surgical intervention of periarticular tissues, including structures containing mechanoreceptors [13], which may be important for joint afferent input. Consistent with this concept, a previous study has reported impairments in balance and postural control after THA [14]. Therefore, greater awareness of the operated hip during daily activities may be related to not only discomfort or reduced functional confidence but also incomplete recovery of sensorimotor control after THA. Importantly, the association between lower FJS at 3 months and subsequent falls remained significant after adjustment for postoperative pain, muscle strength, and gait abnormality, which are established physical risk factors for falls after THA. This suggests that FJS may capture aspects of postoperative recovery that are not fully reflected by conventional clinical measures. Whereas pain, muscle strength, and observable gait characteristics primarily describe physical function, FJS reflects the patient experience and perception of the operated hip during daily activities. Taken together, these findings indicate that early joint awareness may represent a distinct dimension of postoperative recovery that is relevant to fall risk. However, because the exact timing of falls within the 3- to 12-month postoperative interval was unknown, the temporal relationship between changes in joint awareness and fall events could not be determined in detail. Therefore, the proposed attentional resource mechanism should be interpreted as a hypothesis rather than a confirmed explanation.

Falls may be associated with lower FJS at later follow-up after THA. Poorer FJS at 12 months may reflect changes in patient activity behavior during recovery. After experiencing a fall, patients may become more attentive to the operated hip and consciously protect it during daily activities. Consequently, they may be more likely to reduce the use of the operated side, which may be associated with lower FJS at later follow-up.

These findings have potential clinical implications. Conventional fall risk assessment commonly includes clinical factors such as gait and balance impairment, fall history, and physical function, including muscle strength [35]. The FJS-12 reflects the patient’s perception of the operated hip during daily activities and may complement these conventional assessments by providing additional patient-reported information. Further prospective studies, including interventional studies, are needed to determine whether assessment of FJS-12 can improve postoperative fall risk management after THA.

Conversely, fall occurrence between 3 and 12 months was associated with lower FJS-12 at 12 months, even after adjustment for FJS-12 at 3 months. The magnitude of this association may be clinically relevant because the adjusted difference of 14.07 points exceeded the previously reported minimal clinically important difference of 8.1 points for the FJS after THA. However, this comparison should be interpreted cautiously because the reported minimal clinically important difference was derived from a UK population at 6 months postoperatively using patient satisfaction as the anchor [36]. In addition, because the exact timing of falls within the 3- to 12-month interval was unknown, we could not determine whether falls contributed to lower FJS-12 or whether patients with already lower FJS-12 were more likely to experience falls. Therefore, this finding should not be interpreted as evidence that falls caused lower FJS-12. Rather, it should be viewed as hypothesis-generating and may indicate that patients who experience falls after THA represent a subgroup with less favorable recovery trajectories or persistent joint awareness. Future prospective studies that record the timing and circumstances of falls are needed to clarify this relationship.

This study has two major strengths. First, few longitudinal studies have examined the association between joint awareness and falls after THA, evaluating both the relationship between early postoperative FJS and subsequent falls and the association between fall occurrence and recovery of joint unawareness. By evaluating these relationships in both directions, this study provides a comprehensive view of the relationship between joint awareness and fall events during postoperative recovery. Second, the analyses were performed in a relatively large cohort with standardized follow-up at defined postoperative time points and complete data for all variables included. Moreover, multiple clinically relevant confounders, including pain, quadriceps muscle strength, and gait abnormality, were adjusted for, supporting the robustness of the observed associations.

However, some limitations should be acknowledged. First, fall occurrence was assessed using retrospective patient self-report at postoperative follow-up visits. This approach may have introduced recall bias and underreporting, particularly for minor or noninjurious falls [37,38]. In addition, patients with greater joint awareness or less favorable postoperative recovery may have been more likely to recall or report fall events, which could have influenced the observed association. Similarly, although no fractures or other serious fall-related injuries were documented, this finding should be interpreted cautiously because fall-related injuries were not collected prospectively or systematically. Furthermore, falls occurring within the first 3 months after THA were not evaluated because the primary exposure, FJS-12, was assessed at 3 months. Therefore, the present findings do not address fall risk during this early postoperative period. Second, even within the 3- to 12-month interval evaluated in this study, the exact timing of falls was unknown. Therefore, the temporal relationship between changes in joint awareness and fall events could not be determined in detail. This limitation is particularly relevant to the secondary analysis examining the association between fall occurrence and FJS-12 at 12 months, because FJS-12 was not assessed at the time of the fall. Thus, the lower FJS-12 observed at 12 months among patients who experienced falls may reflect poorer overall postoperative recovery, rather than a direct effect of the fall. Third, residual confounding by unmeasured factors cannot be excluded. Although we adjusted for several clinically relevant variables, including pain intensity, quadriceps muscle strength, and gait abnormality, preoperative fall history was not systematically recorded and could not be included in the models. This is an important limitation because prior fall history is a well-established predictor of future falls [35,39]. Other potentially relevant factors, including preoperative physical activity, assistive device use, delirium, frailty, analgesic and psychotropic medication use, and implant-related variables, were also unavailable. Additionally, patients with neurological disorders affecting gait or balance control were excluded, although milder or undiagnosed neurological conditions may not have been fully captured. Depression was identified from medical records rather than by a standardized screening instrument [40], which may have resulted in underascertainment and incomplete adjustment for depression as a confounder. Furthermore, fear of falling or falls self-efficacy was not assessed using a validated fall-related instrument, such as the Falls Efficacy Scale-International, and therefore could not be included as a potential confounder. Consequently, we were also unable to directly compare the FJS-12 with an established fall-related measure. Fourth, this study was conducted at a single institution with a predominantly female Japanese cohort, which may limit generalizability to other populations. The cohort’s demographic characteristics, including a high proportion of women and relatively low body mass index, as well as the distinct etiology of hip osteoarthritis in Japan [41,42], may differ from those in Western THA populations. Therefore, caution is needed when applying these findings to other settings. In addition, 185 eligible patients were excluded from the final analytical cohort. Although we compared the characteristics of included and excluded participants in Supplementary Table 1, some imbalances were observed, particularly in age and contralateral hip condition. Therefore, selection bias cannot be completely excluded. Finally, because of the observational design of this study, causal relationships between joint awareness and falls cannot be established. Although associations between early FJS-12, subsequent falls, and later FJS-12 were observed, these findings should be interpreted as associations rather than evidence of causality.

## Conclusions

Lower FJS at 3 months after THA was associated with a higher risk of subsequent falls and fall occurrence between 3 and 12 months was associated with lower FJS at 12 months. Because the timing of falls during this interval was unknown and residual confounding cannot be excluded, the secondary finding should be interpreted as hypothesis-generating rather than causal. Early postoperative joint awareness may provide additional patient-reported information when assessing fall risk after THA, but future prospective studies are needed to validate whether incorporating FJS-12 into postoperative follow-up can help identify patients who may benefit from further fall-risk assessment or preventive support.

## Declaration of generative AI and AI-assisted technologies in the writing process

During the preparation of this work, the authors used ChatGPT (OpenAI) in order to improve the language and readability of the manuscript. After using this tool/service, the authors reviewed and edited the content as needed and take full responsibility for the content of the publication.

## CRediT authorship contribution statement

**Osamu Wada:** Writing – original draft, Project administration, Methodology, Investigation, Formal analysis, Conceptualization. **Koutatsu Nagai:** Writing – review & editing, Methodology, Formal analysis, Conceptualization. **Masataka Igeta:** Writing – review & editing, Methodology, Formal analysis. **Akiko Mori:** Writing – review & editing. **Kiyonori Mizuno:** Writing – review & editing, Project administration, Investigation.

## Conflicts of interest

The authors declare no potential conflicts of interest.

For full disclosure statements refer to https://doi.org/10.1016/j.artd.2026.102151.

## Figures and Tables

**Supplementary Table 1:** Characteristics of included and excluded participants

Characteristics	Included participants		Excluded participants other than lost follow-up		Lost follow-up participants		SMD^a^	SMD^b^
	N = 623		N = 145		N = 40			
	Mean (SD)	Median [IQR]	Mean (SD)	Median [IQR]	Mean (SD)	Median [IQR]		
Age, year	70.8 (9.4)	71.0 [65.0–77.0]	66.3 (8.0)	66.0 [61.0–72.0]	66.6 (8.9)	66.0 [61.5–72.0]	0.52	0.46
Sex							0.09	0.59
Men	92 (14.8)		17 (11.7)		16 (40.0)			
Women	531 (85.2)		128 (88.3)		24 (60.0)			
BMI	23.9 (3.9)	23.5 [21.1–26.1]	23.7 (4.2)	23.5 [21.0–25.7]	24.6 (3.1)	25.0 [23.1–26.6]	0.06	0.20
Hip condition in the contralateral side								
Total hip arthroplasty	59 (9.4)		2 (1.4)		3 (7.5)		0.36	0.07
Osteoarthritis, no	492 (79.0)		26 (17.9)		20 (50.0)		1.54	0.64
Osteoarthritis, yes	72 (11.6)		117 (80.7)		17 (42.5)		1.93	0.74
Knee arthroplasty, yes	28 (4.5)		3 (2.1)		2 (5.0)		0.14	0.02
Depression, yes	9 (1.4)		2 (1.4)		1 (2.5)		0.01	0.08
Pain, point	1.3 (1.8)	0.0 [0.0–2.0]	1.0 (1.6)	0.0 [0.0–2.0]	1.6 (2.2)	1.0 [0.0–2.5]	0.17	0.15
Quadriceps muscle strength, Nm/kg	1.1 (0.4)	1.0 [0.8–1.3]	1.1 (0.3)	1.0 [0.9–1.4]	1.1 (0.3)	1.1 [0.9–1.3]	0.07	0.16
Gait abnormality, yes	44 (7.1)		16 (11.0)		2 (5.0)		0.14	0.09
FJS-12, point								
3 months	60.8 (23.4)	62.5 [45.8–77.3]	64.1 (22.7)	63.6 [47.9–80.4]	59.6 (25.3)	61.4 [46.7–75.0]	0.14	0.05

IQR, interquartile range; SD, standard deviation; SMD, absolute standardized mean difference.

Values are presented as mean (SD), median [IQR], or n (%). SMDs were calculated as the difference between the groups. An absolute SMD ≥0.10 was used as a descriptive reference for between-group differences. The FJS-12 ranged from 0 to 100, with higher scores indicating lower awareness of the joint during daily activities.

a SMD was calculated between the included participants and the excluded participants other than lost follow-up to describe between-group difference.

b SMD was calculated between the included participants and the lost follow-up participants to describe between-group difference.

**Supplementary Table 2:** Characteristics in fallers

Characteristics	One fall		Two falls		Three falls	
	N = 86		N = 11		N = 2	
	Mean (SD)	Median [IQR]	Mean (SD)	Median [IQR]	Mean (SD)	Median [IQR]
Age, year	71.7 (10.0)	73.0 [65.0–78.0]	74.9 (9.7)	75.0 [66.0–83.0]	76.0 (0)	76.0 [76.0–76.0]
Sex						
Men	10 (11.6)		1 (9.1)		0 (0)	
Women	76 (88.4)		10 (90.9)		2 (100)	
BMI	24.2 (3.8)	23.6 [21.6–26.4]	26.3 (5.3)	24.6 [22.2–32.8]	24.2 (2.5)	24.2 [22.4–26.0]
Hip condition in the contralateral side						
Total hip arthroplasty	7 (8.1)		1 (9.1)		1 (50.0)	
Osteoarthritis, no	68 (79.1)		9 (81.8)		1 (50.0)	
Osteoarthritis, yes	11 (12.9)		1 (9.1)		0 (0)	
Knee arthroplasty, yes	2 (2.3)		1 (9.1)		0 (0)	
Depression, yes	2 (2.3)		0 (0)		0 (0)	
Pain, point	1.6 (1.8)	1.0 [0.0–3.0]	1.7 (2.0)	1.0 [0.0–3.0]	0 (0)	0 [0.0–0.0]
Quadriceps muscle strength, Nm/kg	1.0 (0.4)	1.0 [0.8–1.2]	0.7 (0.3)	0.7 [0.5–1.0]	0.8 (0.3)	0.8 [0.6–1.0]
Gait abnormality, yes	8 (10.8)		2 (18.2)		0 (0)	
FJS-12, point						
3 months	56.0 (23.1)	55.7 [39.6–72.7]	36.4 (26.8)	37.5 [10.0–61.4]	42.6 (28.1)	42.6 [22.7–62.5]
12 months	56.7 (24.9)	56.3 [39.6–75.0]	43.4 (13.2)	42.5 [35.4–56.3]	60.3 (14.5)	60.3 [50.0–70.5]

IQR, interquartile range; SD, standard deviation.

Data are presented as mean (SD) or median [IQR] for continuous variables.

The FJS-12 ranged from 0 to 100, with higher scores indicating lower awareness of the joint during daily activities.

**Supplementary Table 3:** Association between the number of falls between 3 months and 12 months postoperatively and the FJS-12 at 3 months postoperatively

Variables	IRR	95% confidence interval		*P* value
		Lower	Upper	
FJS-12 at 3 months postoperatively, per 10-point decrease	1.16	1.06	1.26	<.001
Age, year	1.02	0.996	1.04	.125
Sex, men	0.86	0.47	1.57	.627
BMI	1.02	0.97	1.07	.414
Depression, yes	1.59	0.55	4.60	.394
THA in the contralateral side, yes	1.02	0.51	2.04	.957
OA in the contralateral side, yes	0.89	0.51	1.56	.689
Pain at 3 months postoperatively	0.93	0.83	1.05	.235
Quadriceps muscle strength at 3 months postoperatively	0.41	0.21	0.83	.012
Gait abnormality, yes	1.20	0.65	2.24	.558

BMI, body mass index; IRR, incidence rate ratio; OA, osteoarthritis.

Covariates were included in the model for adjustment purposes and were not the primary variables of interest.

## Appendix A Supplementary data

Conflict of Interest Statement for Igeta

Conflict of Interest Statement for Mizuno

Conflict of Interest Statement for Mori

Conflict of Interest Statement for Nagai

Conflict of Interest Statement for Wada

STROBE
