# Supplementary material for: Association Between Early Joint Awareness and Falls After Total Hip Arthroplasty
Source: Arthroplast Today. 2026 Nov 4;42:102151. doi: 10.1016/j.artd.2026.102151 (PMC13756305; doi:10.1016/j.artd.2026.102151)
Supplement: Supplementary file 1 [file mmc1.pdf]

INDIVIDUAL CONFLICT OF INTEREST STATEMENT

American Association of Hip and Knee Surgeons

(Adopted from the American Academy of Orthopaedic Surgeons disclosure statement)

The following form **must be filled out completely and submitted by each author (example, 6 authors, 6 forms). All items require a response. If there is no relevant disclosure for a given item, enter "*None*."**

Association Between Early Joint Awareness and Falls After Total Hip Arthroplasty

Manuscript Title

1. Royalties from a company or supplier (The following conflicts were disclosed)
   None

2. Speakers bureau/paid presentations for a company or supplier (The following conflicts were disclosed)
   Paid presentation as a statistical expert for Ono Pharmaceutical Co., Ltd.

3A. Paid employee for a company or supplier (The following conflicts were disclosed)
   None

3B. Paid consultant for a company or supplier (The following conflicts were disclosed)
   Statistical Consultant/Advisor, Takeda Pharmaceutical Company Limited
   Statistical Consultant/Advisor, Santen Pharmaceutical Co., Ltd.
   Statistical Consultant/Advisor, emol Inc.

3C. Unpaid consultants for a company or supplier (The following conflicts were disclosed)
   None

4. Stock or stock options in a company or supplier (The following conflicts were disclosed)
   None

5. Research support from a company or supplier as a Principal Investigator (The following conflicts were disclosed)
   None

6. Other financial or material support from a company or supplier (The following conflicts were disclosed)
   None

7. Royalties, financial or material support from publishers (The following conflicts were disclosed)
   None

8. Medical/Orthopaedic publications editorial/governing board (The following conflicts were disclosed)
   None

9. Board member/committee appointments for a society (The following conflicts were disclosed)
   Editorial Committee Member, Japanese Society of Applied Statistics
   Planning and Public Relations Committee Member, Society for Health Data Science
   Biostatistics Committee Member, Japan Children's Cancer Group (JCCG)

Each author must sign AND print or type his/her name, date and submit a separate form

In addition, one BLINDED Conflict of Interest form (no author names used) should be submitted per manuscript with all author disclosures.

Masataka Igeta	Masataka Igeta	March 20, 2026
Author Name (Print or Type)	Author Signature	Date
